# Identification and Evaluation of Strain B37 of *Bacillus subtilis* Antagonistic to Sapstain Fungi on Poplar Wood

**DOI:** 10.1155/2014/149342

**Published:** 2014-10-21

**Authors:** XiaoHua Zhang, GuiHua Zhao, DeWei Li, ShunPeng Li, Qing Hong

**Affiliations:** ^1^Key Laboratory of Agricultural Environmental Microbiology, Ministry of Agriculture, College of Life Sciences, Nanjing Agricultural University, Nanjing, Jiangsu 210095, China; ^2^Jiangsu Polytechnic College of Agriculture and Forestry, Jurong, Jiangsu 212400, China; ^3^The Connecticut Agricultural Experiment Station Valley Laboratory, 153 Cook Hill Road, Windsor, CT 06095, USA

## Abstract

Devaluation of poplar products by sapstain accounts for huge and unpredictable losses each year in China. We had isolated four poplar sapstain fungi, *Ceratocystis adiposa* Hz91, *Lasiodiplodia theobromae* YM0737, *L. theobromae* Fx46, and *Fusarium* sp. YM05, from five poplar varieties and 13 antagonistic bacteria from nine diverse varieties. After being experimented with agar plates, wood chips, and enzyme activities, strain B37 was identified as the best poplar sapstain biocontrol bacterium. The strain B37 was identified as *Bacillus subtilis* using sequences of the 16S rRNA gene, physiological biochemical, and morphological characteristics.

## 1. Introduction

Hartig [1] confirmed that wood discoloration was caused by a dark mycelial fungus. It was the start of wood stain fungi research. Since then, a large number of studies on stain fungi had been carried out. The molds caused sapstain of pine, kumquat, grapevine,* Abies balsamea* (Linn.) Mill.,* Picea mariana* (Mill.) B.S.P., and so forth include species of the genera* Ceratocystis *Ellis & Halst,* Ophiostoma piceae *(Münch) Syd. & P. Syd.,* Diplodia* Fr.,* Lasiodiplodia* Ellis & Everhart, and so forth [2–4]. These molds caused a wide spectrum of sapwood discoloration including black, red, violet, orange, and green [5]. The predominant wood discoloring species belonged to Ascomycota, which included* Lasiodiplodia*,* Ceratocystis*,* Diplodia*,* Alternaria* Nees, and* Cladosporium* Link [4, 6].

Sapstain and mold growth on lumber were the serious problems in the wood process industry [7]. Biodiscoloration caused by molds in the initial stage of wood decay was one of the most serious discolorations. It caused serious damage to wood, which not only existed on the surface of the wood, but also penetrated wood into its deep interior [8, 9]. Chapman and Scheffer [10] proved that blue stain fungi (*Ceratostomella pilifera* (Fr.) G. Winter,* C. pini* Münch,* C. ips* Rumbold,* Graphium rigidum* (Pers.) Sacc.) could discolor* Pinus echinata* Mill.,* P. ponderosa* Dougl. ex Laws., and* P. taeda* L. The toughness reduced from 9% to 75%; specific gravity losses reached from 1.4% to 3.8%, respectively. The discoloration was objectionable to buyers and highly detrimental to the pulp and paper industry.

In the USA, estimates suggested that about ten million dollars were spent on chemical control each year to prevent sapstain fungi from colonizing the sapwood of sawn lumber, such as methylene bisthiocyanate [11–14]. Behrendt [15] proved that treating the ends of freshly cut timber with the nonpigmented strain of* Ophiostoma piliferum* (Fr.) Syd. and P. Syd. and spraying logs with Dursban 4E could reduce growth of blue stain fungi* Phanerochaete gigantea* (Fr.) S. S. Rattan to 23% in red pine and* Pinus resinosa* Ait. Other chemicals, such as pentachlorophenol, methyl bromide, 3-iodo-2-propynyl butyl carbamate, copper-8-quinolinolate, vinclozolin, iprodione, and procymidone, have been used as fungicides in pine, oak, and poplar [16–21].

Because toxic chemicals may cause secondary pollution in the environment and accumulation in animal tissues, many researchers used various biological methods to inhibit the growth of sapstain [22]. Biological control of sapstain fungi (*Sphaeropsis sapinea* (Fr.) Dyko & B. Sutton (syn.* Diplodia pinea* (Desm.) J. Kickx f.),* Ophiostoma minus*,* Ophiostoma floccosum* Math.-Käärik, and* O. ips* (Rumbold) Nannf.) has been investigated in laboratory trials on wood blocks of* Pinus *sp. using several strains of fungi, such as* Trichoderma* sp.,* Trichothecium roseum* (Pers.) Link and* Phlebiopsis gigantea* (Fr.) Jülich [23, 24]. Morin et al. [25] discovered that a spontaneous albino strain,* Ceratocystis resinifera* Kasper, has the ability to prevent discoloration of spruce sapwood caused by wild-type sapstain fungi. Some genera of bacteria (i.e.,* Bacillus*,* Pseudomonas*,* Lactobacillus*, and* Streptomyces*) also have been investigated in laboratory experiments or field trails to control sapstain fungi [24, 26–32]. Forty-five fungal isolates were tested for the sensitivity to Bacillus subtilis C186. Only* Phialophora richardsiae* 616E appeared unaffected by the* Bacillus* [30]. The results showed that* Bacillus subtilis* C186 had a wide variety of inhibitory effects on sapstain fungi. However, the report about biocontrol of sapstain fungi in poplar is seldom.

The objectives of our study were to screen bacterial strains for their antagonistic activity against the discoloration of poplar wood and to compare the efficiency of different bacteria in preventing sapstain under the same conditions.

## 2. Materials and Methods

### 2.1. Materials

Discolored sapwood of four poplar hybrids was collected from Feixian City, Shandong Province, and Jurong, Siyang, and Suining Cities, Jiangsu Province, between April 2006 and October 2008. Four sapstain fungal strains,* Ceratocystis adiposa* (E. J. Butler) C. Moreau, Hz91 from* Populus deltoides* cv. “Zhonghe” and* Populus* ×* euramericana* cv. “74/76” at Siyang city,* Lasiodiplodia theobromae* (Pat.) Griffon & Maubl., YM0737 and FX46 from* P. deltoides *cl. “Zhonglin-46” and* P. deldoides* Bartr. cv. “Lux” (I-69/55) at Feixian and Suining cities,* Fusarium lateritium* Ness, YM05 from* P. deltoides* cv. “harvard” ×* P. deltoides* cv. “Lux” cv. “Nanlin351” at Jurong City, were isolated by the Center of Biotechnology in Jiangsu Polytechnic College of Agriculture and Forestry, Jiangsu Province, China.

### 2.2. Methods

#### 2.2.1. Screening for Bacteria for Biocontrol against Poplar Discoloration Fungi

**Figure 1:**
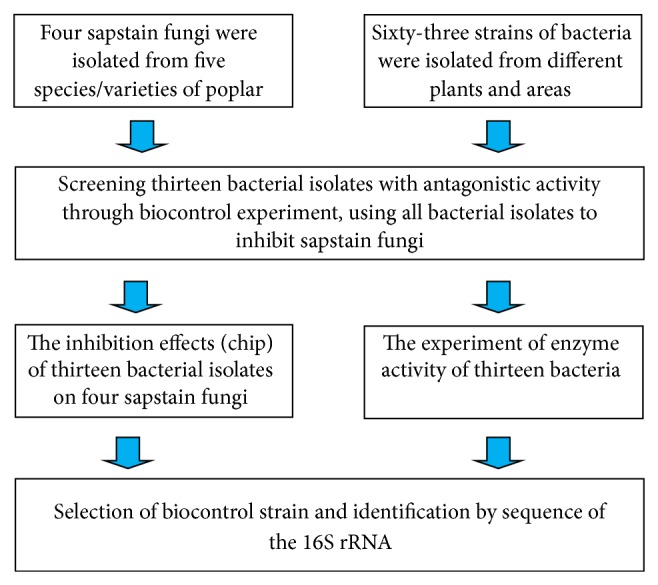
Selection method of biocontrol bacteria for poplar discoloration.

The method used to select and identify bacteria for biocontrol against poplar discoloration fungi was shown in Figure 1.

#### 2.2.2. Isolation of Biocontrol Bacteria

Healthy and diseased timber, twigs and leaves of* Populus* ×* euramericana* (Dode) Guineir cv. “San Martino” (I-72/58),* Populus* ×* euramericana* CL “74/76,”* Populus deltoides* cv. “Zhonghe,”* Camellia oleifera* Abel,* Magnolia grandiflora* Linn,* Phoebe neurantha* Gamble,* Pinus thunbergii* Parl.,* Photinia* ×* fraseri*, were collected from Linyi and Heze Cities, Shandong Province, and Jurong, Siyang, and Shuyang Cities, Jiangsu Province, between February 2009 and December 2010. The materials washed with 70% alcohol for five minutes were cut into blocks (3 × 3 × 3 mm). Every sample was divided into 40–60 blocks which were transferred to Petri dishes with Luria-Bertani (LB) medium (tryptone, 10 g; yeast extract, 5 g; NaCl, 10 g; add deionized H_2_O to 950 mL; adjust the pH to 7.0 with 5 N NaOH. Adjust the volume to 1 liter with deionized H_2_O. It was prepared with 15 g L^−1^ agar. Medium was sterilized at 121°C for 30 min). The plates were incubated for 7 days in dark at 28 ± 1°C and RH 70% in a PRC-250A intelligent artificial climate incubator (Zhejiang, China).

#### 2.2.3. Screening for Antifungal In Vitro Activity

**Figure 2:**
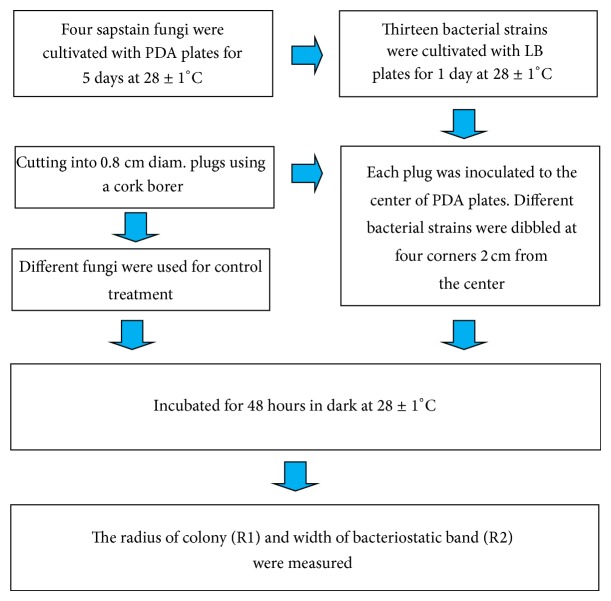
Method of screening for antifungal in vitro activity.

The method of screening for antifungal in vitro activity was shown in Figure 2. Four species of sapstain fungi were cultivated with PDA plates for 5 days at 28 ± 1°C and cut into 0.8-cm diameter plugs using a cork borer, respectively. Each plug was inoculated to the center of PDA plates. Different bacterial strains were dibbled at four corners 2 centimeters from the center. All treatments were conducted in quintuplicate. Different fungi (Hz91, YM05, YM0737, and Fx46) were used for control treatment. The cultures were incubated for 48 hours in dark at 28 ± 1°C and RH 70% in a PRC-250A intelligent artificial climate incubator. The radius of colony (R1) and width of bacteriostatic band (R2) were measured. The ratio of R2 to R1 represented bacteriostatic intensity.

#### 2.2.4. Biocontrol Experiment of Chip

Thin chips (5 × 2 × 0.6 cm in size) of* Populus* ×* euramericana* (Dode) Guineir cv. “San Martino” (I-72/58) were sterilized for 30 min at 121°C. LB liquid cultures of 13 bacteria were cultured for 60 hours at 120 rpm/min and 28 ± 1°C. The chips were dipped in bacterial cultures for 1 min, removed, and then air dried. The chip for control experiment was dipped in LB liquid medium for 1 min. Plugs of 0.8 cm diameter of four species of sapstain fungi that were grown on PDA plates for 5 days were used to inoculate the center of chips, respectively. Sterilized chips were put on a piece of filter paper over vermiculite in Petri dishes. The treated chips were cultivated separately for 4, 8, and 12 days at 28 ± 1°C for observing results.

#### 2.2.5. Screening for Enzyme Activity

Thirteen bacteria were inoculated on casein, chitin, or beta-glucan extract agar in Petri dishes according to the method of Cota et al. [33]. All treatments were conducted in quintuplicate and all plates were incubated at 28 ± 1°C. The color change of the medium was observed after 2 days. The width of color change band represents protease, chitinase, and beta-glucanase activity.

#### 2.2.6. Detailed Identification for Candidate of the Biocontrol Agents

For identification for candidates of the biocontrol agents, the methods of morphological, physiological, and biochemical characteristics as well as molecular biological technology were used. The detailed procedure and methods of physiological and biochemical tests of the candidate were according to the Manual of Systematic and Determinative Bacteriology [34] and Bergey's Manual of Determinative Bacteriology, 9th edition [35]. Gram-stain of the candidate was observed under an optical compound microscope ZEISS Imager A1 (Carl Zeiss, Jena, Germany) (*n* = 30). The morphology of cells was examined by transmission electron microscopy (Hitachi, Tokyo, Japan). Bacterial cells cultured for 10 hours for electron microscopy were picked up using an inoculating loop and dipped in a drop of water to make suspension. The suspension was transferred to carbon-coated copper grids with a capillary pipet. After a short time, excess liquid was absorbed with a piece of filter paper from the edge of the copper grids. Cells were negatively stained with 2% phosphotungstic acid for 90 seconds. A piece of filter paper was used to absorb extra dye. The sample was air dried and examined.

#### 2.2.7. PCR Amplification and Sequencing of 16s rRNA

Total DNA was extracted and purified according to the procedure described by Devereux and Willis [36]. Agarose (0.7%) gel electrophoresis was used to verify DNA quality. The following universal primers were used to amplify the nearly complete 16s rRNA. The forward primer was 5′-AGAGTTTGATCCTGGCTCAG-3′, and the reverse primer was 5′-GCCTTGTACACACCGCCC-3′ [37]. The total volume (25 *μ*L) of the PCR reaction mixture contained 2.5 *μ*L of 10 × Taq buffer, 1.5 *μ*L of MgCl_2_ (25 mmol L^−1^), 2 *μ*L of each deoxyribonucleoside triphosphate (10 mmol L^−1^), 1 *μ*L of each primer (30 pmol), 1 *μ*L of DNA template, 0.5 *μ*L of Taq DNA polymerase, and 15.5 *μ*L of H_2_O. The PCR reaction was performed using a GeneAmp PCR System 9700 (Applied Biosystems, Shanghai, China) with the following thermal program: an initial denaturation step (94°C, 5 min) followed by 30 cycles of denaturation (95°C, 30 s), annealing (54°C, 30 s), and extension (72°C, 60 s). After a final extension step (72°C, 20 min), samples were kept at 4°C. The GenScript Co., Ltd. (Nanjing, China) sequenced the 16s rRNA gene. The 16S rRNA of the thirteen bacteria was aligned with published sequences from the GenBank database using Bioedit 7.0 alignment tool comparison software.

### 2.3. Data Analysis

Statistical analysis of the results was performed using the SPSS13.0 analytical software.

## 3. Results and Analysis

### 3.1. Selection of Biocontrol Bacteria

Sixty-three strains of bacteria (*Bacillus*,* Pseudomonas*,* Enterobacter*, and* Streptomyces*) were isolated from different plants, but 19 strains showed antagonistic activity to four poplar discoloration fungi (Table 1). Thirteen strains were selected for further investigation.  Table 2 showed that* Bacillus* and* Pseudomonas* were the predominant genus, and* Bacillus subtilis* and* Bacillus amyloliquefaciens* were the dominant species in 13 strains. The results were similar to the study of Seifert et al. [30].

### 3.2. Experiment of Biocontrol

The antagonistic activity of 13 bacteria to 4 species of poplar sapstain fungi was showed in Table 3. The results showed that strains B82 and B37 had strongest inhibition of Hz91, YM05, YM0737, and FX46. The inhibition of strains B4 and B1 to four fungi was the worst. Strain B9 antagonizes only YM05. Strain B123 had inhibitory effect on the fungus Hz91. The ratio of R2 to R1 of strain B82 to Hz91 and YM05 is 1.35 and 1.89, respectively. The ratio of R2 to R1 of strain B37 to YM0737 is 1.33. Antagonistic activity of strains B82 and B37 to FX46 is similar; the ratio of R2 to R1 is 1.41 and 1.40, respectively. Statistical analysis of these results showed no significant difference (*P* < 0.05) for Hz91, YM05, and FX46 between strain B82 and strain B37. Significant difference (*P* < 0.05) between strain B82 and strain B37 was for YM0737.

The results of the chip experiment were showed in Table 3. The strains of B4, B9, B33, B123, and B1 had no antagonistic effect on the 4 species of poplar sapstain fungi. The strains of B37 and B82 expressed antagonistic effect. FX46, YM05, and Hz91 were inhibited for 12 days by B37, whereas YM0737 was inhibited only for 8 days. The strain of B82 had antagonistic effect on FX46 and YM05 for 12 days, but on Hz91 for 8 days and on YM0737 for 4 days.

The enzymes activity of 13 bacteria was showed in Table 3. The results showed that all 13 bacteria did not produce chitinase. Strain B37 was the best bacterium to produce *β*-glucanase; the width of color change band is 4.25 cm. There was only a little amount of *β*-glucanase produced by strains B33 and B13; the width of color change band is 0.29 cm and 0.38 cm, respectively. Strains B82, B8, and B9 showed a better activity to produce protease than those of strains of B47, B27, B4, B30, and B37. The width of color change band is 4.13 cm by strain B82. Other bacteria did not produce protease. Analysis of enzymes activity showed a significant difference (*P* < 0.05) between strain B82 and strain B37.

In experiment of antifungal in vitro activity, thirteen strains were proved to inhibit 4 sapstain fungi. Nine strains (B8, B13, B27, B33, B37, B47, B82, B123, and B140) of them are* Bacillus*. Three strains (B4, B9, and B30) of them belong to* Pseudomonas*. One (B1) is* Streptomyces*. The ratio of R2 to R1 of strain B82 to Hz91, YM05, YM0737, and FX46 is 1.35, 1.89, 1.00, and 1.41, respectively. The ratio of R2 to R1 of strain B37 to Hz91, YM05, YM0737, and FX46 is 0.99, 1.48, 1.33, and 1.40, respectively. In experiment of chip, YM05 and FX46 were biocontrolled by B37 and B82 for 12 days. However, strain of B37 can inhibit Hz91 and YM0737 for 12 and 8 days, respectively. Strain of B82 can inhibit Hz91 and YM0737 for 8 and 4 days, respectively. So strain B37 of* Bacillus subtilis* was a better biocontrol bacterium than B82 on poplar sapstain (*C. adiposa* Hz91,* L. theobromae* YM0737,* L. theobromae* Fx46, and* Fusarium* sp., YM05).

### 3.3. Morphological Characteristics of Strain B37

**Figure 3:**
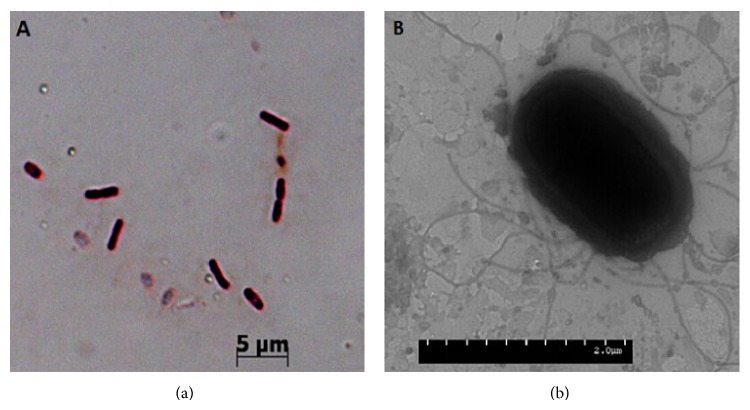
Morphology of* Bacillus subtilis* strain B37. (a) Rod-shaped cells of strain B37. (b) Flagella on a bacterial cell of strain B37 (the photo was taken with TEM at the Life Science Laboratory Center of Nanjing Agriculture University, China).

Strain B37 was Gram-positive rod-shaped with blunt ends (Figure 3(a)). On LB medium, colony was larger, milky white, of 3~5 mm diameter, after training 24 hours, with the raised surface being dry and wrinkled. The size was 1.83~2.41 × 0.56–1.02 *μ*m, with an average of 2.1 × 0.74 *μ*m with peritrichous flagella (Figure 3(b)). Endospore was located in the middle of the cell and the size was 0.62~1.08 × 0.44~0.66 *μ*m with an average of 0.86 × 0.51 *μ*m.

### 3.4. Physiological and Biochemical Characteristics of Strain B37

According to the Manual of Systematic and Determinative Bacteriology [34] and Bergey's Manual of Determinative Bacteriology, 9th edition [35], results of tests on physiological and biochemical characteristics of strain B37 identified strain B37 as* Bacillus subtilis* (Ehreberg) Cohn (Table 4). The results showed that treatment of indole reaction, gas production, and lactose have no effect, and the other 18 treatments have positive effect.

### 3.5. The 16S rRNA of Strain B37 Sequencing and Phylogenetic Tree

**Figure 4:**
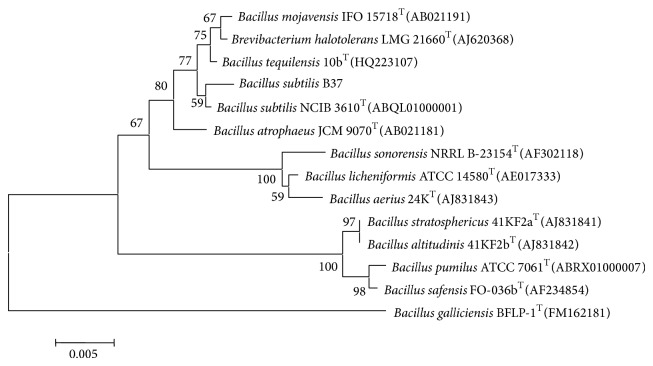
A neighbor-joining phylogenetic dendrogram based on 16S rRNA gene sequences showing the position of strain B37 among members of the genus* Bacillus* species. Numbers on branch nodes are percentage bootstrap values (100 resamplings). The “T” in the figure means “type strain.”

The results of the 16S rRNA of strain B37 sequencing and aligning with published sequences from the GenBank database demonstrated that strain B37 had a 99.3% similarity to* Bacillus subtilis* NCIB 3610^T^ (GenBank accession number ABQL01000001) (CP000560) (Figure 4). According to morphology, physiological and biochemical characteristics, and 16S rRNA sequence, strain B37 was identified as* Bacillus subtilis* and 16S rRNA of strain B37 was deposited at GenBank under accession number JN656409. Strain B37 was storing at China General Microbiological Culture Collection Center (CGMCC number 4277).

## 4. Discussion

Sapstain fungi are economically important and have been studied for many years in different countries. The major sapstaining groups belong to the* Aureobasidium*,* Ceratocystis*,* Ophiostoma*,* Sporothrix*,* Graphium*, or* Hyalorhynocladiella* and* Leptographium* genera [4, 38].

In our work, we used four sapstain fungi (*C. adiposa *Hz91,* L. theobromae *YM0737 and FX46,* and F. lateritium *YM05) isolated from discolored poplar as pathogen.* Lasiodiplodia theobromae* which can cause death of the kumquat (*Fortunella margarita* (Lour.) Swingle) tree was isolated from tissue taken from the margin of discolored kumquat bark and wood on symptomatic branches in orchards in Taiwan [2]. Various studies confirmed that* L. theobromae* was the causal agents of dieback and canker of grapevines in northern Mexico and* Pinus caribaea* var.* hondurensis* and* Citrus aurantifolia* in Venezuela [3, 39]. It also caused discoloration of rubberwood and heveawood in tropical countries [40, 41]. The ascomycete* Ceratocystis resinifera* is a pathogen of* Picea* and* Pinus* tree or freshly felled spruce and pine logs in Continental Europe, North America, Norway, and Canada [4, 42, 43].* Ceratocystis coerulescens* (Münch) B. K. Bakshi can stain Jack pine, white spruce, lodgepole pine, and Corsican pine in Canada [44].* Fusarium *spp. is one kind of Sapstain fungi from* Pinus radiata* D. Don in Korea [45]. However, the study on stain to poplar was scarce.

Strain B37 of* Bacillus subtilis* was the best biocontrol bacterium on poplar sapstain (*C. adiposa* Hz91,* L. theobromae* YM0737,* L. theobromae* Fx46, and* Fusarium* sp., YM05) of the 13 bacteria in our work. It was isolated from normal leaf of* Pinus thunbergii*. These observations agree with Feio et al. [46], who reported that the growth of* Fusarium oxysporum *was inhibited by* B. subtilis* 335 on yeast extract glucose broth (YGB) medium on days 11 and 14. Growth of* F. oxysporum* was significantly inhibited by 50% solution of YGB inoculated with 11-day-old* B. subtilis* 335 culture in blocks of maritime pine (*Pinus pinaster*). The 12-h yeast malt (YM) broth medium of* B. subtilis* C186 can prevent the growth of several fungi, isolated from* Pinus* spp.,* Aureobasidium* sp.,* Ceratocystis* sp.,* Cytospora pini* Desm*., Ophiostoma* sp.,* Penicillium* sp., and* Trichocladium* sp. [30]. However, very few studies were biocontrol poplar sapstain by* B. subtilis*.

Chitinase and *β*-l,3-glucanase that degrade the fungal cell wall components, chitin and *β*-l,3-glucan, are important enzymes to control fungi (*Fusarium solani* (Mart.) Sacc.,* Neurospora crassa* Shear & B. O. Dodge,* Rhizoctonia solani* J. G. Kühn,* Sclerotium rolfsii* Sacc., and* Pythium ultimum* Trow) [47, 48]. Cultivated for 2 days, strain B37 produced a significant amount of *β*-1,3-glucanase (the width of color change band is 4.25 cm), effectively inhibiting the growth of four poplar sapstain fungi. It can produce a small amount of proteases (the width of color change band is 0.76 cm), but chitinase was not found. *β*-1,3-Glucanase can catalyze the hydrolysis of *β*-1,3-dextran polymers, which inhibit the growth and reproduction of fungi [49]. *β*-1,3-Glucanase genes clearly resulted in resistance to infection by* Fusarium* [50]. Therefore, *β*-1,3-glucanase is one of the important inhibitions of the plant fungal disease, so the metabolic mechanism of strain B37 will be the direction of our further study.

## Figures and Tables

**Table 1:** The isolation of biocontrol bacteria from different plants.

Trial	Plants	Isolation localities	Total number of samples	Number of isolates tested	Antagonistic number	Selected for further investigations
1	*Populus* × *euramericana* (Dode) Guineir cv. “San Martino” (I-72/58)	Wood discoloration, bark, bud	56	12	3	3
2	*Populus* × *euramericana* CL “74/76”	Normal branch for 1 year, canker	42	8	3	1
3	*Populus deltoides* cv. “Zhonghe”	canker	47	9	3	2
4	*Camellia oleifera* Abel	Leaf spot	45	6	2	2
5	*Magnolia grandiflora* Linn	Leaf spot	50	10	4	2
6	*Phoebe neurantha* Gamble	Leaf spot	50	5	1	1
7	*Pinus thunbergii* Parl.	Normal leaf	45	4	1	1
8	*Photinia* × *fraseri* red robin	Branch scab and leaf spot	60	9	2	1

Total			395	63	19	13

**Table 2:** The source of 13 biocontrol bacteria.

Strain	Identification	Sample source	Sampling localities
B1	*Streptomyces setonii *	Leaf spot of *Camellia oleifera* Abel	Jurong, Jiangsu Province, China
B4	*Pseudomonas fluorescens *	Leaf spot of *Magnolia grandiflora* Linn	Jurong, Jiangsu Province, China
B8	*Bacillus amyloliquefaciens *	Leaf spot of* Magnolia grandiflora* Linn	Jurong, Jiangsu Province, China
B9	*Pseudomonas fluorescens *	Leaf spot of* Phoebe neurantha* Gamble	Jurong, Jiangsu Province, China
B13	*Bacillus stratosphericus *	Ulcer of* Populus deltoides* cv. “Zhonghe”	Jinan, Shandong Province, China
B27	*Bacillus subtilis *	Bark and wood of *Populus* × *euramericana* (Dode) Guineir cv. “San Martino” (I-72/58)I-72	Siyang and Suining, Jiangsu Province, China
B30	*Pseudomonas fluorescens *	Bud and wood of* Populus* × *euramericana* (Dode) Guineir cv. “San Martino” (I-72/58)I-72	Shuyang, Jiangsu Province, China
B33	*Bacillus methylotrophicus *	Brown patch of* Populus* × *euramericana* (Dode) Guineir cv. “San Martino” (I-72/58)I-72	Siyang, Jiangsu Province, China
B37	*Bacillus subtilis *	Normal leaf of *Pinus thunbergii* Parl.	Jurong, Jiangsu Province, China
B47	*Bacillus subtilis *	Canker and wood of* Populus* × *euramericana* CL “74/76”I-107	Mengyin, Feixian, Yinan, Yiyuan, Junan, Luozhuang, Lanshan, Shandong Province, China
B82	*Bacillus amyloliquefaciens *	Leaf spot of* Photinia* × *fraseri* red robin	Jurong, Jiangsu Province, China
B123	*Bacillus subtilis *	Canker of* Populus deltoides* cv. “Zhonghe”	Feixian, Shandong Province, China
B140	*Bacillus amyloliquefaciens *	Leaf spot of* Camellia oleifera* Abel.	Jurong, Jiangsu Province, China

**Table 3:** Antagonistic activity of 13 bacteria to 4 species of poplar sapstain fungi.

Strain	Antagonistic activity^a^				Experiment of chip^b^												Enzymes activity (mm)		
	Hz91	YM05	YM0737	FX46	Hz91			YM05			YM0737			FX46			Proteases	Chitinases	Beta- glucanases
					4 d	8 d	12 d	4 d	8 d	12 d	4 d	8 d	12 d	4 d	8 d	12 d			
B82	1.35 ± 0.18^A^ < ?ehlt?>	1.89 ± 0.18^A^	1.00 ± 0.17^B^	1.41 ± 0.20^A^	+	+	—	+	+	+	+	—	—	+	+	+	41.30 ± 0.62^A^	—	—
B47	0.77 ± 0.06^ABC^	1.83 ± 0.13^A^	—	0.52 ± 0.14^CD^	—	—	—	+	—	—	—	—	—	—	—	—	14.70 ± 0.44^D^	—	—
B33	0.22 ± 0.08^CD^	0.65 ± 0.05^BC^	0.56 ± 0.07^CD^	0.54 ± 0.11^CD^	—	—	—	—	—	—	—	—	—	—	—	—	—	—	2.90 ± 0.42^C^
B140	0.64 ± 0.17^BC^	1.50 ± 0.01^AB^	1.00 ± 0.10^B^	0.70 ± 0.22^BCD^	+	+	—	+	—	—	—	—	—	—	—	—	—	—	—
B30	0.72 ± 0.20^BC^	0.88 ± 0.13^ABC^	0.49 ± 0.08^CD^	0.39 ± 0.09^CDE^	+	—	—	—	—	—	—	—	—	—	—	—	8.60 ± 0.32^F^	—	—
B13	0.68 ± 0.15^BC^	1.63 ± 0.24^AB^	0.63 ± 0.13^C^	1.30 ± 0.28^A^	—	—	—	+	—	—	—	—	—	+	—	—	—	—	3.80 ± 0.55^B^
B8	1.19 ± 0.13^A^	1.49 ± 0.14^AB^	0.54 ± 0.12^CD^	0.84 ± 0.12^BC^	+	—	—	+	—	—	—	—	—	—	—	—	36.20 ± 1.66^B^	—	—
B9	—	0.98 ± 0.19^ABC^	—	—	—	—	—	—	—	—	—	—	—	—	—	—	27.50 ± 0.95^C^	—	—
B4	0.29 ± 0.03^CD^	0.27 ± 0.02^C^	0.24 ± 0.07^DE^	0.35 ± 0.11^DE^	—	—	—	—	—	—	—	—	—	—	—	—	5.30 ± 0.50^G^	—	—
B1	0.11 ± 0.06^D^	0.25 ± 0.13^C^	—	—	—	—	—	—	—	—	—	—	—	—	—	—	—	—	—
B27	0.87 ± 0.13^AB^	1.64 ± 0.20^AB^	0.67 ± 0.07^C^	1.10 ± 0.26^AB^	—	—	—	+	—	—	—	—	—	+	—	—	13.10 ± 0.56^E^	—	—
B37	0.99 ± 0.08^AB^	1.48 ± 0.16^AB^	1.33 ± 0.19^A^	1.40 ± 0.22^A^	+	+	+	+	+	+	+	+	—	+	+	+	7.60 ± 0.76^F^	—	42.50 ± 0.95^A^
B123	0.98 ± 0.12^AB^	—	—	—	—	—	—	—	—	—	—	—	—	—	—	—	—	—	—

^a^R2/R1 represent the antagonistic activity; —, no antagonism; values in a column followed by the same letter are not significantly different according to Duncan test (*P* < 0.05).

^b^+, antagonism; —, no antagonism.

**Table 4:** Physiological and biochemical characteristics of strain B37.

Treatment	Results
Oxidase	+
Catalase	+
Starch hydrolysis	+
Nitrate reduction	+
Gelatin liquefaction	+
V-p	+
Indole reaction	—
Glucose	
Acid production	+
Gas production	—
Lactose	—
Methyl red	+
Citric acid	+
Pectin	+
Sucrose	+
Maltose	+
Xylose	+
Galactose	+
Mannose	+
Fructose	+
Glycerin	+
Urease	+

+, positive effect; —, no effect.
